# A Reversible Colorimetric and Fluorescence “Turn-Off” Chemosensor for Detection of Cu^2+^ and Its Application in Living Cell Imaging

**DOI:** 10.3390/molecules24234283

**Published:** 2019-11-25

**Authors:** Yun Hu, Aiqian Chen, Zhuo Kong, Demeng Sun

**Affiliations:** Department of Bioengineering, Zhuhai Campus, Zunyi Medical University, Zhuhai 519041, China; caq0620@163.com (A.C.); kz1825278000@163.com (Z.K.); m15713712002@163.com (D.S.)

**Keywords:** copper ion, chemical sensor, 3-hydroxyflavone

## Abstract

Dual-function chemosensors that combine the capability of colorimetric and fluorimetric detection of Cu^2+^ are still relatively rare. Herein, we report that a 3-hydroxyflavone derivative (*E*)-2-(4-(dimethylamino)styryl)-3-hydroxy-4*H*-chromen-4-one (**4**), which is a red-emitting fluorophore, could serve as a reversible colorimetric and fluorescence “turn-off” chemosensor for the detection of Cu^2+^. Upon addition of Cu^2+^ to **4** in neutral aqueous solution, a dramatic color change from yellow to purple-red was clearly observed, and its fluorescence was markedly quenched, which was attributed to the complexation between the chemosensor and Cu^2+^. Conditions of the sensing process had been optimized, and the sensing studies were performed in a solution of ethanol/phosphate buffer saline (*v*/*v* = 3:7, pH = 7.0). The sensing system exhibited high selectivity towards Cu^2+^. The limit of naked eye detection of Cu^2+^ was determined at 8 × 10^−6^ mol/L, whereas the fluorescence titration experiment showed a detection limit at 5.7 × 10^−7^ mol/L. The complexation between **4** and Cu^2+^ was reversible, and the binding constant was found to be 3.2 × 10^4^ M^−1^. Moreover, bioimaging experiments showed that **4** could penetrate the cell membrane and respond to the intracellular changes of Cu^2+^ within living cells, which indicated its potential for biological applications.

## 1. Introduction

Copper is an essential transition element in the human body that plays a critical role in many biological processes. High concentrations of copper can be found in the liver and brain [1]. Generally, the copper iron acts as a catalytic cofactor for a variety of metalloenzymes, including cytochrome oxdidase, superoxide dismutase, and tyrosinase, etc. [2]. However, excessive uptake of copper would cause liver or kidney damage and is associated with various neurodegenerative diseases, such as Alzheimer’s disease and Wilson’s disease [3,4]. Copper is normally found as Cu(II) in water, and the permissible maximum level in drinking water is set at 3 × 10^−5^ mol/L by the World Health Organization (WHO) [5]. Yet, due to the increasing discharge of metal waste from industry and agriculture, copper has been identified as an environmental pollutant [6], so it is highly important to develop sensitive and selective methods for the detection of Cu^2+^ in environmental and biological samples.

Traditional analytical methods for the detection of Cu^2+^ include electrochemical methods (potentiometry, voltammetry, etc.), inductively coupled mass spectroscopy, atomic absorption spectroscopy, etc. [7,8]. However, there are plenty of shortcomings in these methods because they are usually complicated, time-consuming, and costly. In recent years, increased attention has been given to the development of colorimetric and fluorimetric methods, which exhibit advantages of low cost, simplicity, convenience, and high sensitivity [9,10,11]. The colorimetric method allows simple naked-eye detection without the use of any expensive equipment, and the fluorimetric method has the ability of real-time analysis and biological imaging [12]. Both of the methods mostly employ a chemosensor that undergos a specific interaction with Cu^2+^ [1,13]. Obviously, compared to the single-functional one, a chemosensor that combines the capability of colorimetric and fluorimetric detection will be more desirable [1,2,14]. Though considerable efforts have been made, dual-functional chemosensors for detection of Cu^2+^ are still relatively rare.

The 3-hydroxyflavones are the known molecules that undergo an excited-state intramolecular proton transfer (ESIPT) process [15]. These molecules often exhibit a large Stokes shift upon irradiation and possess many favorable optical properties, such as good photophysical stability and reasonable fluorescent quantum yields [16,17]. Plenty of biochemical chemosensors have been developed based on their molecular scaffold [15,18,19]. However, to the best of our knowledge, the 3-hydroxyflavones have never been used for the study of sensing Cu^2+^ before. Herein we report that a 3-hydroxyflavone derivative (*E*)-2-(4-(dimethylamino)styryl)-3-hydroxy-4*H*-chromen-4-one (**4**), which is a red-emitting fluorophore, could serve as a reversible colorimetric and fluorescence “turn-off” chemosensor for detection of Cu^2+^. The sensing of Cu^2+^ is rapid and highly sensitive in neutral aqueous solution. In addition, **4** might be further used in monitoring the intracellular changes of Cu^2+^ in living cells.

## 2. Results

### 2.1. Optimum Conditions for Sensing Process

#### 2.1.1. Effect of Solution

**Figure 1:**
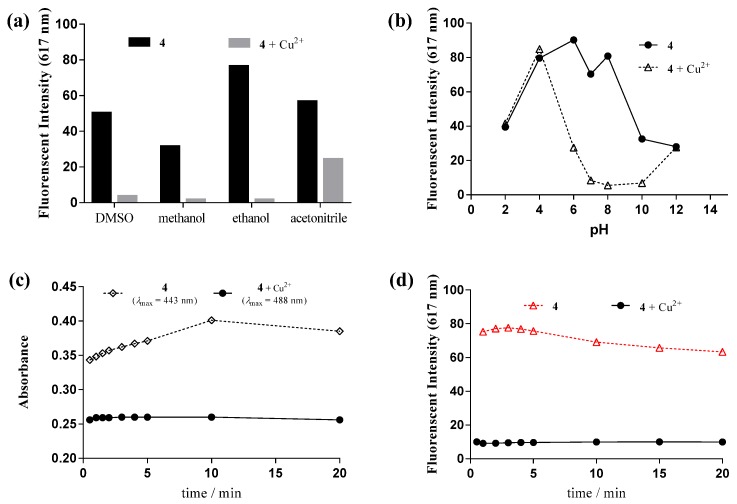
Optimum conditions of 3-hydroxyflavone derivative (*E*)-2-(4-(dimethylamino)styryl)-3-hydroxy-4*H*-chromen-4-one (**4**) for the sensing of Cu^2+^. (**a**) Fluorescence intensities at 617 nm of 20 μM of **4** upon addition of 1 equiv. Cu^2+^ in various organic solvents with phosphate buffer saline (PBS) (*v*/*v* = 3/7, pH = 7.0). (**b**) Effects of pH on the fluorescence intensities at 617 nm of 20 μM of **4** with or without addition of 1 equiv. Cu^2+^ in ethanol/CH_3_COOH-CH_3_COO^−^ solution (*v*/*v* = 3/7). Absorbance (**c**) and fluorescent (**d**) kinetics data of 20 μM of **4** in the absence or presence of 20 μM Cu^2+^ in ethanol/PBS (*v*/*v* = 3/7, pH = 7.0) solution.

The sensing process can be affected by the aqueous environment in which the complexation takes place. Therefore, it is necessary to find a suitable medium for the sensing of Cu^2+^. For this purpose, various water-miscible organic solvents were evaluated as the reaction solution. The changes of their fluorescence spectra were analyzed after the addition of Cu^2+^. As shown in Figure 1a, compared to dimethyl sulfoxide (DMSO), methanol, and acetonitrile the best fluorescence quenching efficiency was obtained in ethanol, and the optimal proportion with PBS (pH = 7.0) is 3:7 (Figure S4). Furthermore, in such solution the complexation took place very quickly (within 1 min), and the formed product remained stable for at least 20 min (Figure 1c,d). These results indicated that the solution of ethanol/PBS (*v*/*v* = 3:7) was suitable for sensing and was used in all the experiments here presented, unless otherwise stated.

#### 2.1.2. Effect of pH

The pH value of a solution is another key factor that affects the sensing process. CH_3_COOH/CH_3_COO^−^ buffer solutions of various pH were prepared and mixed with ethanol, in which the fluorescence response of the chemosensor was analyzed after the addition of Cu^2+^. It was found that the fluorescence intensity of **4** and its sensing of Cu^2+^ were pH sensitive. As shown in Figure 1b, high fluorescent quenching efficiencies were obtained over the pH range 6–8, yet no considerable fluorescence changes were observed over other pH ranges. These results suggests that **4** could perform the sensing of Cu^2+^ in neutral or near neutral conditions and may be further used for biological applications, so all of the following experiments were performed in a neutral solution (pH = 7.0), unless other way stated.

### 2.2. UV-Vis Measurement and Visual Detection

**Figure 2:**
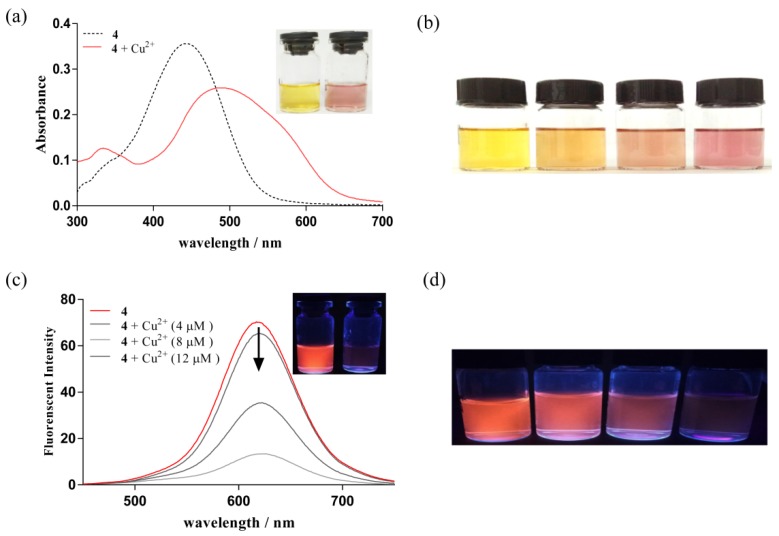
(**a**) Absorption spectra of **4** (20 μM) in the absence or presence of Cu^2+^ (20 μM). Inset: the color changes of **4** (left) upon addition of Cu^2+^ (right) under daylight; (**b**) Visible color changes of **4** in the presence of various concentrations of Cu^2+^ (0, 4, 8, 12 μM); (**c**) Fluorescence spectra of **4** (20 μM) in the absence or presence of Cu^2+^ (0, 4, 8, 12 μM). Inset: the fluorescence changes of **4** (left) upon addition of 20 μM Cu^2+^ (right) under UV light (λ = 365 nm); (**d**) Fluorescence changes of **4** in the presence of various concentrations of Cu^2+^ (0, 4, 8, 12 μM).

The UV-Vis spectra of **4** was first investigated in the presence of Cu^2+^. As shown in Figure 2a, the free **4** exhibited a strong absorption band at 443 nm. Upon addition of Cu^2+^, the band disappeared and a new broad absorption band with a red-shift of 45 nm was observed, which indicated that a new stable complex product was formed. In the daylight, the process exhibited a distinct color change from yellow to purple-red, which was clearly visible to the naked eye. Further, to determine the limit of visual detection, we had monitored the color changes of **4** upon the addition of various concentrations of Cu^2+^. As illustrated in Figure 2b, **4** showed a limit of visual detection for Cu^2+^ at 8 × 10^−6^ mol/L.

### 2.3. Fluorescence Measurements

We then analyzed the fluorescence spectra of **4** with the addition of Cu^2+^. As shown in Figure 2c,d, the free **4** (20 μM) exhibited a red fluorescence with a maximum emission centered at 617 nm. However, the fluorescence was gradually quenched when an incremental amount of Cu^2+^ was added.

**Figure 3:**
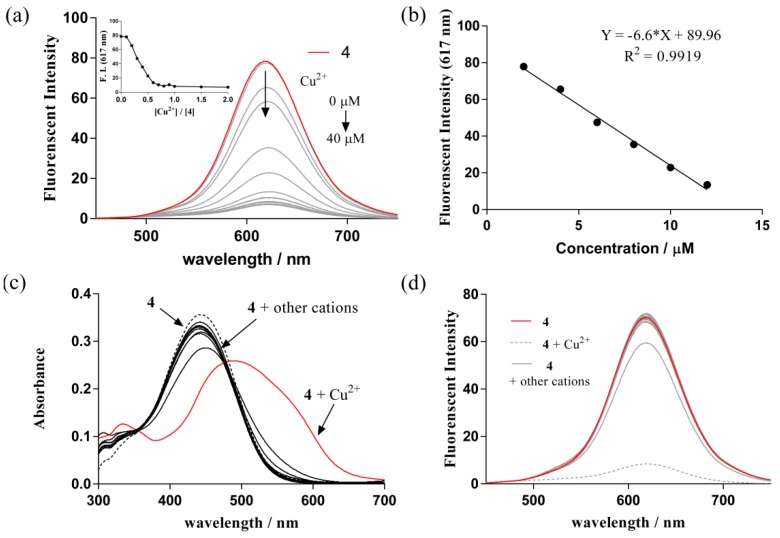
(**a**) Fluorescence titration of **4** upon the addition of Cu^2+^ (0–40 μM). Inset: The fluorescence intensity curve of **4** at 617 nm; (**b**) The linear fluorescence intensity of **4** against the concentrations of Cu^2+^; Absorption spectra (**c**) or fluorescence spectra (**d**) of **4** in the presence of various metal cations (20 μM).

We further performed the fluorescence titration experiment. As shown in Figure 3a, when the addition of Cu^2+^ exceeded 1 equiv. (20 µM), a decreased saturation curve of fluorescence intensity was observed (see inset of Figure 3a). The fluorescence quenching efficiency was 90% when 20 µM Cu^2+^ was added (the quenching efficiency was calculated as (*I*_0_ − *I*)/*I*_0_ × 100; *I*_0_ was the intensity of free **4**, *I* was the intensity of 4 upon the addition of Cu^2+^), which was supposed to be attributed to the paramagnetic nature of Cu^2+^ [20]. In the concentration range of 2–12 μM, when Cu^2+^ was added, a good linear relationship of emission intensity (617 nm) versus the concentration of Cu^2+^ was observed (*R*^2^ = 0.9919, y = 89.96 – 6.6x) (Figure 3b). According to the International Union of Pure and Applied Chemistry (IUPAC) definition [20,21,22], the limit of detection (LOD) of **4** was calculated by the following equation: LOD = 3*σ*/*k* (*σ* is the standard deviation of the blank, and *k* is the slope of the calibration curve line). The LOD of **4** towards Cu^2+^ was determined as 5.7 × 10^−7^ mol/L, which is much lower than the permissible maximum level of Cu^2+^ in drinking water. These results suggest that **4** can be potentially employed to detect Cu^2+^ quantitatively using the fluoremetric method.

When the addition of Cu^2+^ exceeded 1 equiv., a decreased saturation curve of fluorescence intensity was observed (see inset of Figure 3a). The binding constant obtained from the Benesi–Hildebrand plot of 1/(*I* − *I_0_*) versus 1/[Cu^2+^] was found to be 3.2 × 10^4^ M^−1^ (Figure S5).

### 2.4. Selectivity of Detection

**Figure 4:**
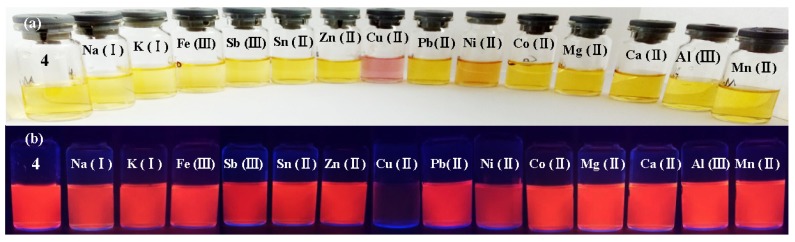
(**a**) Color and (**b**) fluorescent changes of **4** in the presence of various metal ions. **4** was 20 μM, metal ions were 20 μM.

**Figure 5:**
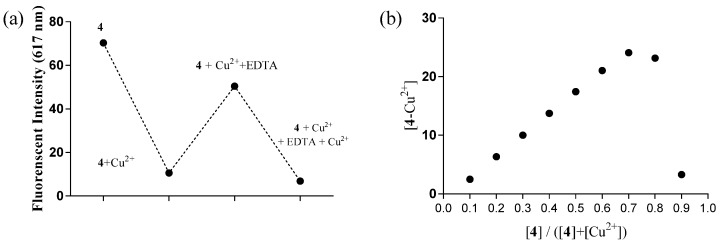
(**a**) Fluorescence intensities at 617 nm of 20 μM of **4** in the presence of Cu^2+^ (20 μM) before or after adding 1 equiv. of EDTA. (**b**) Job’s plot according to the method of continuous variation. The molar ration of **4** changed from 0.1 to 0.9 while keeping the total concentration of **4** and Cu^2+^ at 40 μM.

To evaluate the selectivity of the sensing system, the UV-Vis and fluorescence spectra of **4** were measured in the presence of Cu^2+^ as well as other common metal irons, including Al(III), Ca (II), Co(II), Fe (III), K(I),Mg(II), Mn(II), Na(I),Ni (II), Pb (II), Sn (II), Sb (III), and Zn (II). As shown in Figure 3c–d, upon the addition of metal ions, except Cu^2+^, there were no considerable changes in the optical spectra of **4**, although Ni^2+^ induced a slight effect on its absorption and fluorescence intensity. The color and fluorescence of the chemosensor remained almost unchanged (Figure 4). In addition, an interference study was performed to further investigate the selectivity of the sensing system towards Cu^2+^. As shown in Figure 5, the UV-Vis and fluorescence spectra of 4-Cu^2+^ were hardly affected in the presence of other metal ions (20 μM). These results clearly indicate that **4** exhibits a high sensing selectivity towards Cu^2+^.

### 2.5. Reversibility

The binding reversibility of **4** was estimated with the help of EDTA. As shown in Figure 5a, 85% of the fluorescence intensity of **4** (20 μM) was quenched in the presence of 1 equiv. Cu^2+^. After the addition of EDTA at the same concentration, more than 70% of fluorescence intensity was restored. However, the restored fluorescence was turned off again when adding another 1 equiv. Cu^2+^. These results suggest that the complexation between **4** and Cu^2+^ is reversible, and the chemosensor could be reusable for further analytical tests.

### 2.6. Interaction Mechanism of 4 with Cu^2+^

To study the interaction mechanism of **4** with Cu^2+^, the method of continuous variation (Job’s plot) was first employed. The molar ration of **4** changed from 0.1 to 0.9 while keeping the total concentration of **4** and Cu^2+^ at 40 μM. The concentration of the **4**-Cu^2+^ complex was calculated by the equation: [**4**-Cu^2+^] = *∆I*/*I*_0_ × [**4**] (*I*_0_ was the fluorescent intensity of free **4**, *∆I* = |*I* − *I*_0_|, *I* was the fluorescent intensity of **4** upon the addition of Cu^2+^) [23]. The Job’s plot was plotted between [**4**-Cu^2+^] versus [**4**]. As shown in Figure 5b, the maximum value of [**4**-Cu^2+^] in the Job’s plot appeared at the molar ration of 0.7, which indicated the 2:1 binding stoichiometry between **4** and Cu^2+^.

**Figure 6:**
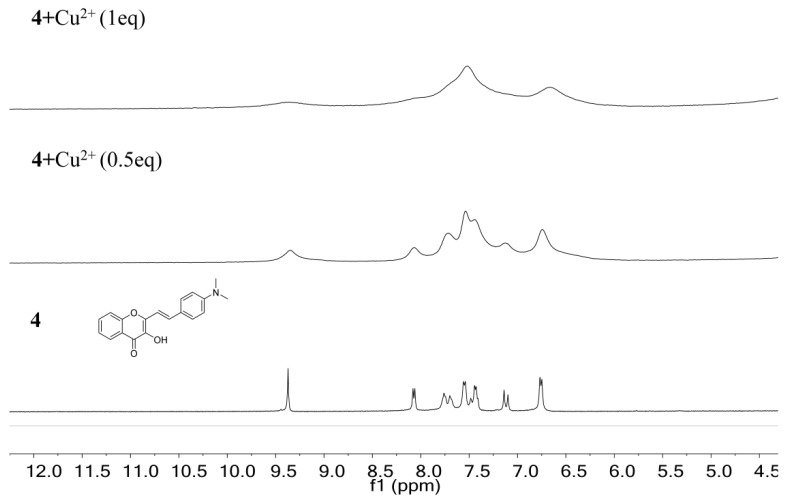
Parts of the ^1^H-NMR spectra of **4** without or with 0.5 and 1 equiv. Cu^2+^.

^1^H-NMR titration experiments were then performed to further investigate the binding mode of **4** with Cu^2+^. As shown in Figure 6, the free chemosensor **4** displayed a sharp peak at 9.37 ppm, which was assigned to the proton of the hydroxyl group. Upon the addition of 0.5 and 1 equiv. Cu^2+^ to the solution of **4** in DMSO-*d*_6_, the peak of the hydroxyl proton was getting broad and almost disappeared, which suggested that the hydroxyl group of **4** was involved in the coordination with Cu^2+^. Peaks of other groups started to combine after the addition of Cu^2+^, indicating that the coordination product has a more rigid and complicated structure [24].

**Scheme 1:**
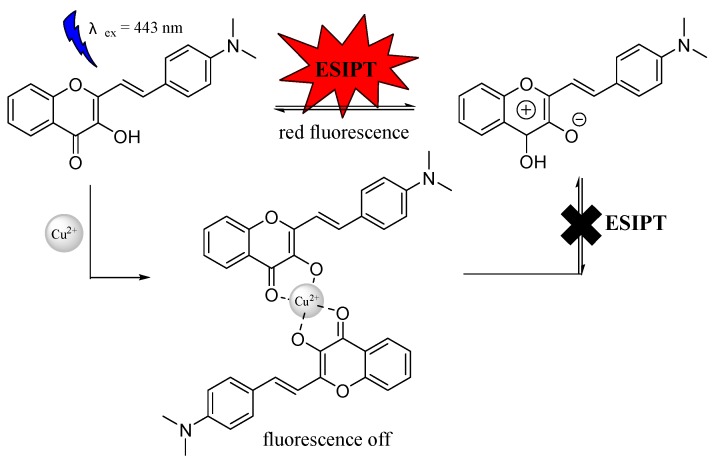
Proposed interaction mechanism of 4 with Cu^2+^. (ESIPT:Excited-state intramolecular proton transfer)

Based on the results from the Job’s plot and ^1^H-NMR titration studies, the possible interaction mechanism between **4** and Cu^2+^ is illustrated in Scheme 1. The fluorescence quenching effect is likely attributed to the coordination with Cu^2+^, which blocks the ESIPT process of the chemosensor, **4**.

### 2.7. Bioimaging Experiments

**Figure 7:**
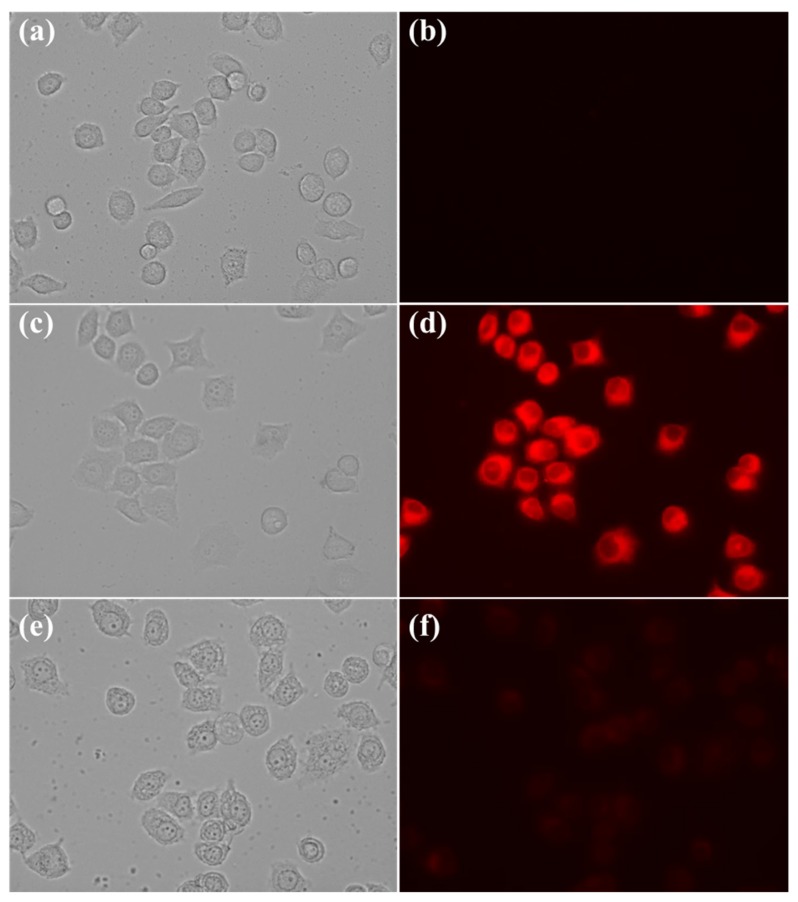
Live-cell imaging of HepG2 cells treated with 0.1% DMSO (**a**,**b**) or 10 μM of **4** before (**c**,**d**) and after incubation with 20 μM of Cu^2+^ (e,f). (**a**,**c**,**e**) represent the bright-field images. (**b**,**d**,**f**) represent the fluorescence images. Images were captured using a 40× objective lens.

Common fluorescent probes of Cu^2+^ show emission within the green spectral region (below 600 nm), which coincides with that of many endogenous chromophores in biological systems, while probes with longer wavelength excitation and emission would bring lower background fluorescence, deeper penetration, and less phototoxicity. Therefore, red-emitting fluorescent probes will be more suitable for bioimaging [25]. To confirm that **4** could perform the sensing of Cu^2+^ in biological systems, we then evaluated its sensing ability in living cells. Because copper tends to accumulate in the liver cells and leads to toxicity [26], we choose the human liver cancer cells HepG2 for the bioimaging experiments. First, to determine the cell permeability of **4**, HepG2 cells were incubated with 10 μM of **4** for 15 min at 37 °C and were then washed with PBS. As shown in Figure 7b, cells treated with vehicle did not show any fluorescence emission, while cells treated with **4** exhibited distinct red fluorescence (Figure 7d). However, after incubation with 20 μM Cu^2+^ for 15 min, the intracellular fluorescence almost disappeared (Figure 7f). These results indicate that **4** is cell-permeable and could respond to the intracellular changes of Cu^2+^.

## 3. Materials and Methods

All commercial reagents and solvents were purchased from vendors and were used without further purification or distillation. Synthetic reactions were monitored by thin-layer chromatography (TLC) on a glass plate coated with silica gel with fluorescent indicator (GF254). Column chromatography was performed on silica gel (200–300 mesh). ^1^H-NMR and ^13^C-NMR spectra were recorded using tetramethylsilane (TMS) as an internal standard with a Burker Biospin Ultrashield 400 NMR system (Rheinstetten, Germany)). High resolution mass spectra (HRMS) were recorded on an Agilent Technologies 6530 Q-TOF (Santa Clara, CA, USA). UV-Vis spectra was recorded using a UV2600 spectrometer (Shimadzu, Japan). Fluorescence spectral studies were performed using an F-4500 fluorescence spectrophotometer (Hitachi, Japan) equipped with a quartz cell of 10 mm path length.

### 3.1. Synthesis of (E)-2-(4-(dimethylamino)styryl)-3-hydroxy-4H-chromen-4-one (**4**)

**Scheme 2:**
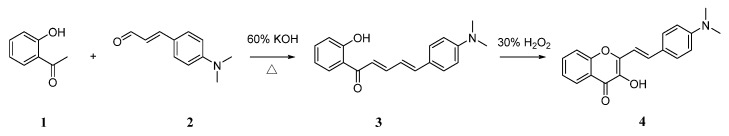
The synthesis of **4**.

The synthesis of **4** is illustrated in Scheme 2. A mixture of 4 mmol (0.54g) 2′-hydroxyacetophenone (**1**), 6 mmol (1.05g) 4-dimethylaminocinnam- aldehyde (**2**), and 4 mL 60% Potassium hydroxide (KOH) in 10 mL ethanol was heated at 60 °C. The reaction progress was monitored by TLC until completed. After being cooled to room temperature, the mixture was diluted with ice cold water and then neutralized with 5% HCl. The precipitate formed was then filtered off and then purified by column chromatography. The intermediate (**3**) was obtained as black powder (0.4g), yield 51%.

Compound **3** (1 mmol, 0.307g) and 20% NaOH (6 mL) were stirred in 10 mL methanol. Then 30% H_2_O_2_ (5 mL) was slowly added under an ice water bath, and the resulting mixture was kept stirred at room temperature for 2 h. When the reaction was completed, the methanol was removed in vacuum and the residue was diluted with water. After being neutralized with 5% aqueous HCl, the mixture was extracted with ethyl acetate three times. The organic layer was combined and washed with excess water and then dried over anhydrous sodium sulfate. After removal of the organic solvent in vacuum, the crude product was purified by column chromatography to give **4** as a red powder in 13.3% yield. ^1^H-NMR (400 MHz, DMSO-*d_6_*) δ 9.37 (s, 1H), 8.07 (d, *J* = 7.6 Hz, 1H), 7.73 (d, *J* = 23.9 Hz, 2H), 7.55 (d, *J* = 7.7 Hz, 2H), 7.50–7.39 (m, 2H), 7.12 (d, *J* = 16.0 Hz, 1H), 6.76 (d, *J* = 7.7 Hz, 2H), 2.99 (s, 6H). (Figure S1, Supplementary Information). ^13^C-NMR (100 MHz, DMSO-*d_6_*) δ 171.84, 154.55, 151.43, 148.02, 137.56, 134.48, 133.64, 125.19, 124.65, 123.81, 122.66, 118.40, 112.57, 110.77. (Figure S2, Supplementary Information). HRMS calculated for C_19_H_17_NO_3_, [M + H]^+^: 308.1281, found 308.1294. (Figure S3, Supplementary Information)

### 3.2. Spectral Measurements

Generally, the stock solutions of **4** were prepared in ethanol. The metal salts, i.e., CuSO_4_, Al(NO_3_)_3_, CoCl_2_, CaCl_2_, FeCl_3_, KCl, MgSO_4_, MnCl_2_, NaCl, NiCl_2_, PbCl_2_, SbCl_3_, SnCl_2_, and ZnCl_2_ were of analytical reagent grade and used without further purification. The stock solutions of metal ions (10 mM) were prepared in distilled water and diluted to the desired concentrations. For the optical measurements, **4** was diluted to 20 μM in an ethanol with phosphate buffer saline (PBS) or CH_3_COOH-CH_3_COO^−^ buffer solution. For the reversibility study, Ethylenediaminetetraacetic acid (EDTA) was heated until completely dissolved in ultrapure water, and the stock solution was prepared at 10 mM. The UV-Vis and fluorescence spectra of the chemosensor in the absence or presence of various guests were recorded at room temperature. The slit size for excitation and emission was 10 nm.

### 3.3. Cell Culture

Human liver carcinoma cell lines (HepG2) were grown in Dulbecco’s modified eagle’s medium (DMEM) containing L-glutamine supplemented with penicillin (100 U/mL), streptomycin (100 ug/mL), and 10% (*v*/*v*) heat-inactivated fetal bovine serum (FBS) in a humidified atmosphere of 5% CO_2_ and 95% O_2_ at 37 °C. First, the cells were incubated with 10 μM of **4** for 15 min at 37 °C. Then the cells were washed with PBS three times and then captured under a fluorescence microscope. Then 2 equivalents of Cu^2+^ were added in the growth medium for 15 min at 37 °C. After being washed with PBS three times, the cells were captured under the fluorescence microscope again. All the pictures were captured using the EVOS FL fluorescence microscope (Thermo Fisher, USA).

## 4. Conclusions

In summary, we have found that the 3-hydroxyflavon derivative **4**, which is a red-emitting fluorophore, could serve as a reversible colorimetric and fluorescence “turn-off” chemosensor for Cu^2+^. In the ethanol-PBS solution (*v*/*v* = 3:7, pH = 7.0), **4** showed a rapid color change from yellow to purple-red upon the addition of Cu^2+^, and its fluorescence was quenched simultaneously. The limit of naked eye detection for Cu^2+^ was observed at 8 × 10^−6^ mol/L, and a lower detection limit was determined as 5.7 × 10^−7^ mol/L from the fluorescence titration experiment, both of which were much lower than the permissible maximum level of Cu^2+^ in drinking water. The sensing of Cu^2+^ by **4** was highly selective and was not influenced even in the presence of other metal ions. Furthermore, **4** was cell-permeable and could respond to the intracellular changes of Cu^2+^, which indicated its potential for biological applications.

## Supplementary Materials

The following are available online: Figure S1: ^1^H-NMR (400 MHz) spectrum of **4** in DMSO-*d_6_*; Figure S2: ^13^C-NMR (100 MHz) spectrum of **4** in DMSO-*d_6_*; Figure S3: HRMS spectrum of **4**. Figure S4: Fluorescence spectra of **4** (20 μM) upon the addition of Cu^2+^ (20 μM) in ethanol/PBS solution of various proportions; Figure S5: The Benesi–Hildebrand plot of 1/(*I* − *I_0_*) versus 1/[Cu^2+^]. *I_0_* was the intensity of free **4**, *I* was the intensity of **4** upon the addition of Cu^2+^.
