# Supplementary material for: A Reversible Colorimetric and Fluorescence “Turn-Off” Chemosensor for Detection of Cu2+ and Its Application in Living Cell Imaging
Source: Molecules. 2019 Nov 25;24(23):4283. doi: 10.3390/molecules24234283 (PMC6930558; doi:10.3390/molecules24234283)
Supplement: Supplementary file 1 [file molecules-24-04283-s001.pdf]

A reversible colorimetric and fluorescence "turn-off" chemosensor for detection of Cu(Ⅱ) and its application in living cell imaging

Yun Hu, Aiqian Chen, Zhuo Kong, Demeng Sun*

Department of Bioengineering, Zhuhai Campus, Zunyi Medical University, Zhuhai 519041, China

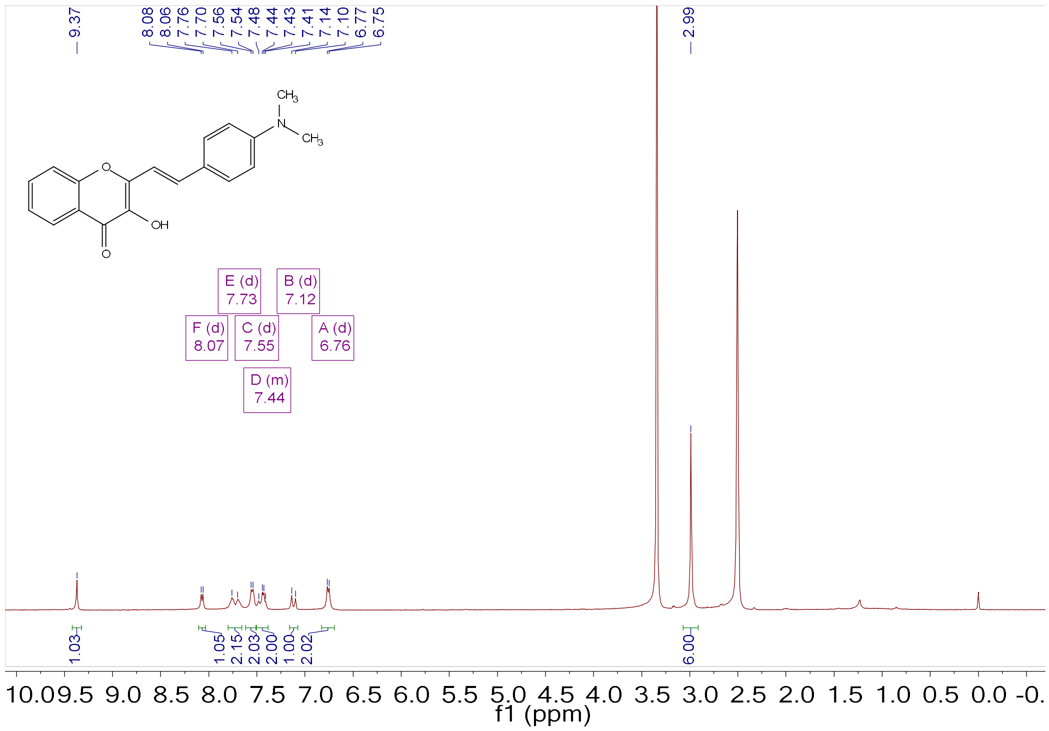

Figure S1: ^{1}H-NMR (400 MHz) spectrum of **4** in DMSO-*d₆*

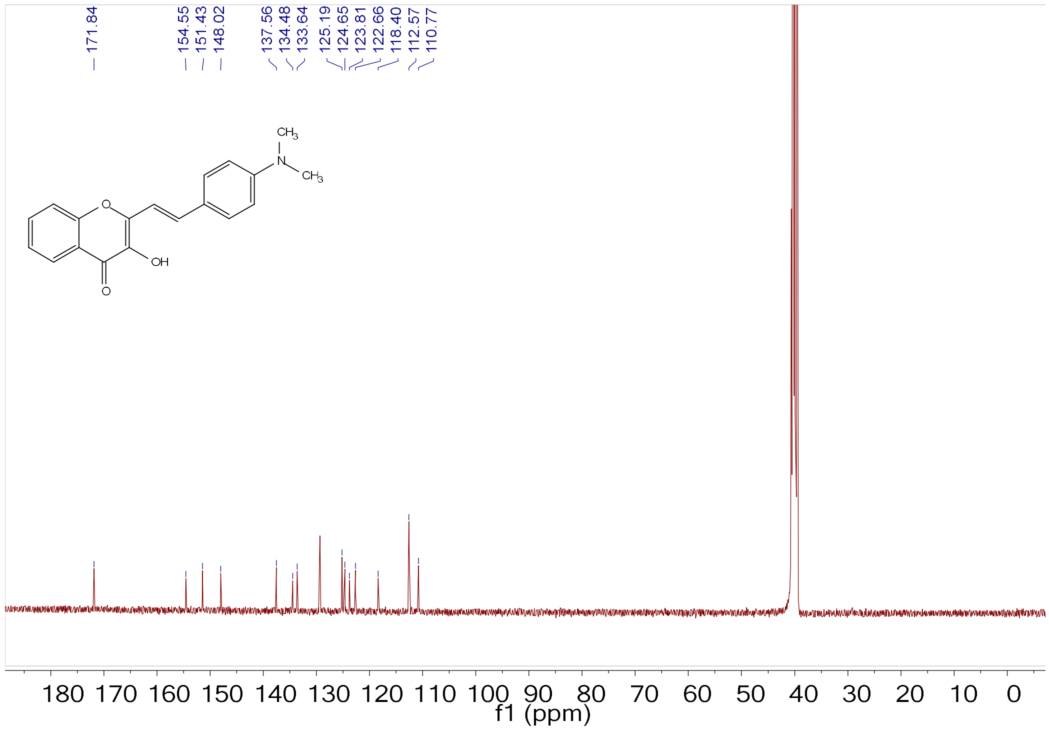

Figure S2: ^{13}C-NMR (100 MHz) spectrum of **4** in DMSO-*d*

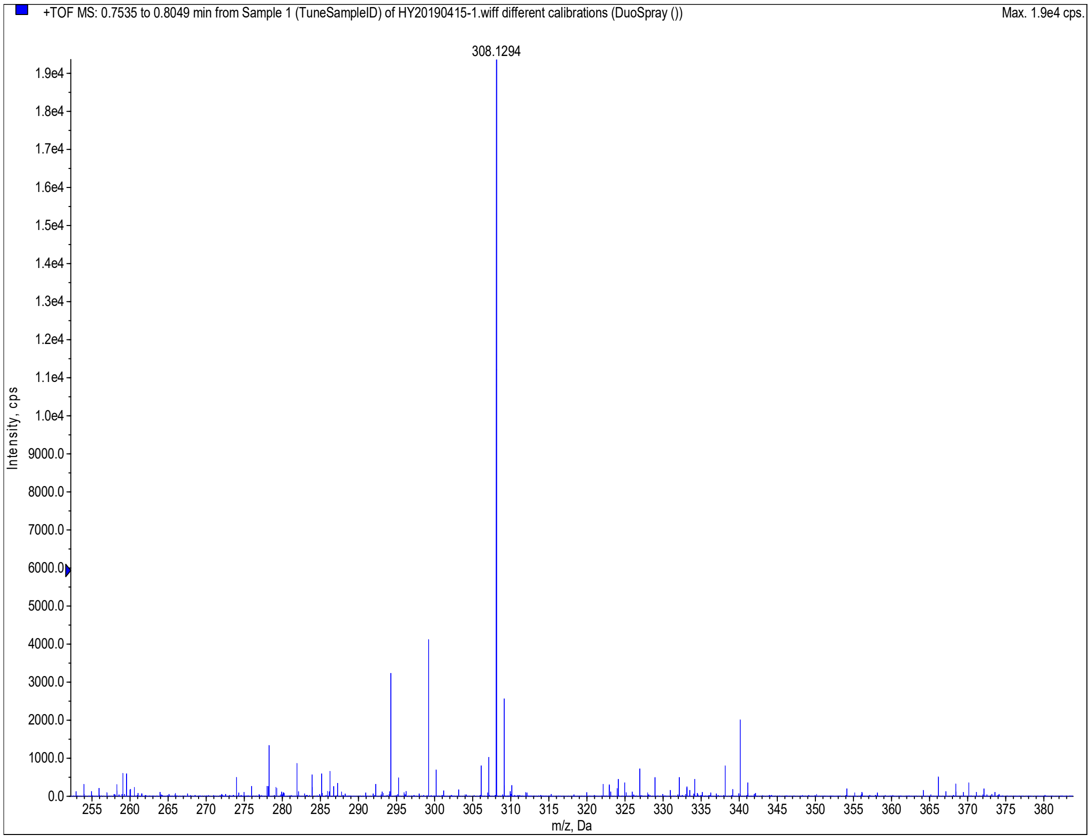

Figure S3: HRMS spectrum of **4**

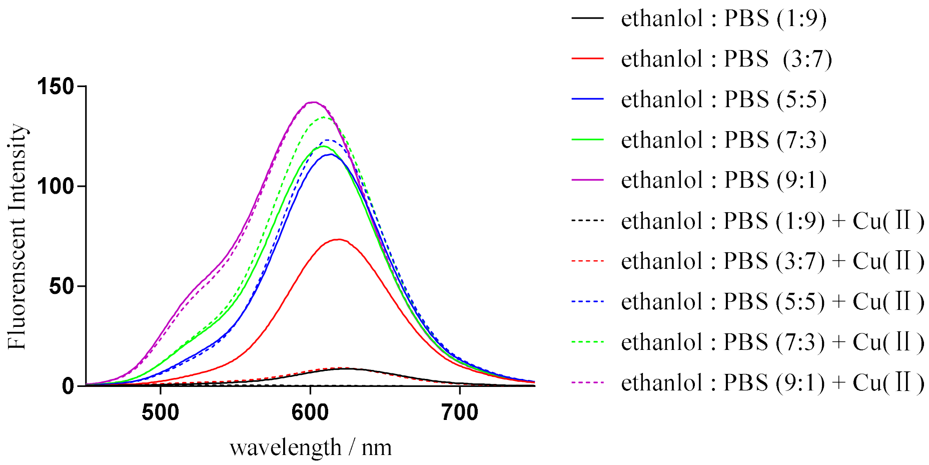

Figure S4: Fluorescence spectra of **4** (20 μM) upon addition of Cu(Ⅱ) (20 μM) in ethanol/PBS solution of various proportions.

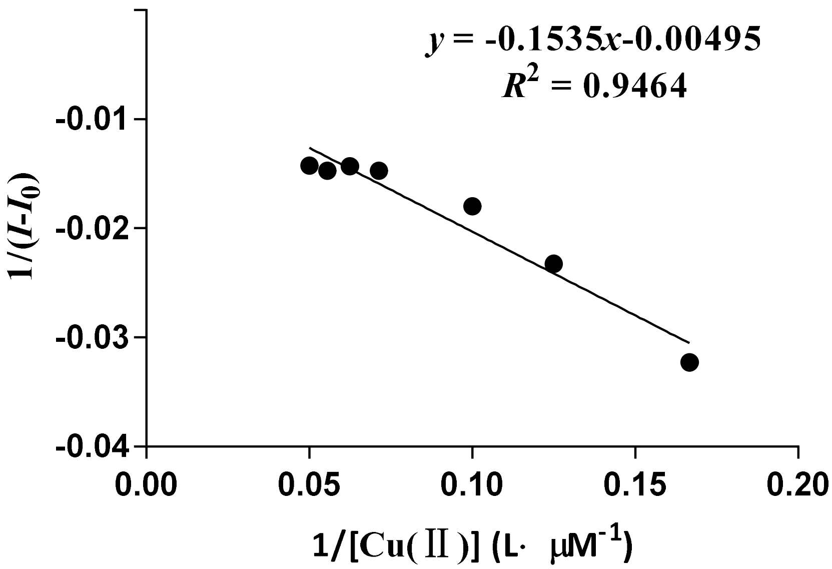

Figure S5: The Benesi-Hildebrand plot of 1/(I-I_0) versus 1/[Cu^{2+}]. I_0 was the intensity of free **4**, I was the intensity of **4** upon addition of Cu^{2+}.
